# Interaction of TAGLN and USP1 promotes ZEB1 ubiquitination degradation in UV-induced skin photoaging

**DOI:** 10.1186/s13578-023-01029-z

**Published:** 2023-05-06

**Authors:** Yinan Li, Xiu Huang, Jing Jin, Haohao Zhang, Kai Yang, Jingxia Han, Ying Lv, Yu Sun, Cheng Yao, Tingting Lin, Caibin Zhu, Huijuan Liu

**Affiliations:** 1 Cheermore Cosmetic Dermatology Laboratory, Shanghai, China; 2 State Key Laboratory of Medicinal Chemical Biology and College of Pharmacy, Nankai University, Tianjin, China; 3 Medical plastic and cosmetic center, Tianjin Branch of National Clinical Research Center for Ocular Disease, Tianjin Medical University Eye Hospital, Tianjin, China

**Keywords:** TAGLN, Zerumbone, ZEB1, Photoaging, Deubiquitination

## Abstract

**Background:**

Ultraviolet A (UVA) irradiation can lead to skin damage and premature skin aging known as photoaging. This work found that UVA irradiation caused an imbalance between dermal matrix synthesis and degradation through the aberrant upregulation of transgelin (TAGLN) and studied the underlying molecular mechanism.

**Results:**

Co-immunoprecipitation and proximal ligation assay results showed that TAGLN can interact with USP1. USP1 can be retained in the cytoplasm by TAGLN in UVA-induced cells, which inhibits the interaction between USP1/zinc finger E-box binding homeobox 1 (ZEB1), promote the ubiquitination degradation of ZEB1, and lead to photoaging. TAGLN knockdown can release USP1 retention and help human skin fibroblasts (HSFs) resist UVA-induced damage. The interactive interface inhibitors of TAGLN/USP1 were screened via virtual docking to search for small molecules that inhibit photoaging. Zerumbone (Zer), a natural product isolated from *Zingiber zerumbet* (L.) Smith, was screened out. Zer can competitively bind TAGLN to reduce the retention of USP1 in the cytoplasm and the degradation of ZEB1 ubiquitination in UV-induced HSFs. The poor solubility and permeability of Zer can be improved by preparing it as a nanoemulsion, which can effectively prevent skin photoaging caused by UVA in wild-type (WT) mice. Zer cannot effectively resist the photoaging caused by UVA in *Tagln*^*−/−*^ mice because of target loss.

**Conclusions:**

The present results showed that the interaction of TAGLN and USP1 can promote ZEB1 ubiquitination degradation in UV-induced skin photoaging, and Zer can be used as an interactive interface inhibitor of TAGLN/USP1 to prevent photoaging.

**Supplementary Information:**

The online version contains supplementary material available at 10.1186/s13578-023-01029-z.

## Background

The long-term exposure of the skin to ultraviolet (UV) rays from the sun causes skin structure and function to deteriorate gradually [1]. Among the three UV rays (UVA, UVB, and UVC), UVA (320–400 nm) penetrates deep into the dermis, then triggers the production of large concentrations of reactive oxygen species (ROS), viz. hydrogen peroxide (H_2_O_2_), free radicals, singlet oxygen, and hyperoxide. These substances cause a variety of chronic skin damage that is related to photoaging, including sagging, slacking, patchy/spotted pigmentation, increased wrinkles, and dryness [2]. However, the mechanism of UVA-induced skin damage has not been fully elucidated.

The transcription factor, zinc finger E-box binding homeobox 1 (ZEB1), is an important member of the zinc finger homeodomain transcription factor family. It can bind with E-box to promote or inhibit molecular transcription and regulate the differentiation and multidrug resistance of cancer cells [3]. Previous studies focused on its role in the epithelial–mesenchymal transition. Studies have shown that EMT and senescence seem to cross paths, with several factors including ZEB1 playing dominant roles in both settings [4]. Studies demonstrated that expression of ZEB1 decreased after UVA irradiation, following a UVA-induced increase of intracellular ROS. This effect causes the increase in the expression of aging-related proteins, such as P53, and therefore leads to cell senescence [1]. ZEB1 may play an essential regulatory role in the aging process. However, the molecular mechanism of UV-induced reduction of ZEB1 remains unclear. ZEB1 is usually degraded by the ubiquitin–proteasome system. Ubiquitination and deubiquitination are reversible post-translational modifications that rely on ubiquitin ligases and deubiquitinases (DUBs) [5]. They are involved in most processes of cell biology, such as protein degradation, DNA replication, and DNA repair [6, 7]. The literature showed that DUBs, such as USP51, USP7, and USP17, can act on ZEB1 [8–10]. The elucidation of the molecular mechanism of the UV-induced decrement in ZEB1 can provide a new idea for finding active small molecules that ameliorate UV-induced skin aging.

Transgelin (TAGLN) is an actin-binding protein that can regulate the dynamics of the actin cytoskeleton and promote the aggregation of G-actin with F-actin [11]. Recent studies revealed that TAGLN expression increases after UV irradiation [12]. TAGLN is an actin-binding protein that affects the dynamics of the actin cytoskeleton, regulating cell migration and movement [13, 14]. However, few studies have investigated whether TAGLN can regulate UV-induced skin aging and whether a relationship exists between TAGLN and ZEB1.

The present work found that the skin aging degree of *Tagln* knockout (*Tagln*^*−/−*^) mice [15] was considerably lower than that of wild-type (WT) mice at the same age. ZEB1 expression decreased whereas TAGLN expression increased in UVA-induced human skin fibroblast (HSF) models. Further research demonstrated that TAGLN can regulate the level of ZEB1 ubiquitination by retaining the deubiquitinase USP1 in the cytoplasm. The interactive interface inhibitor, zerumbone (Zer), was screened out based on the interaction between TAGLN and USP1, providing a small molecular inhibitor for UV-induced photoaging.

## Results

### Skin aging phenotype in *Tagln*^*−/−*^ mice was alleviated

**Fig. 1:**
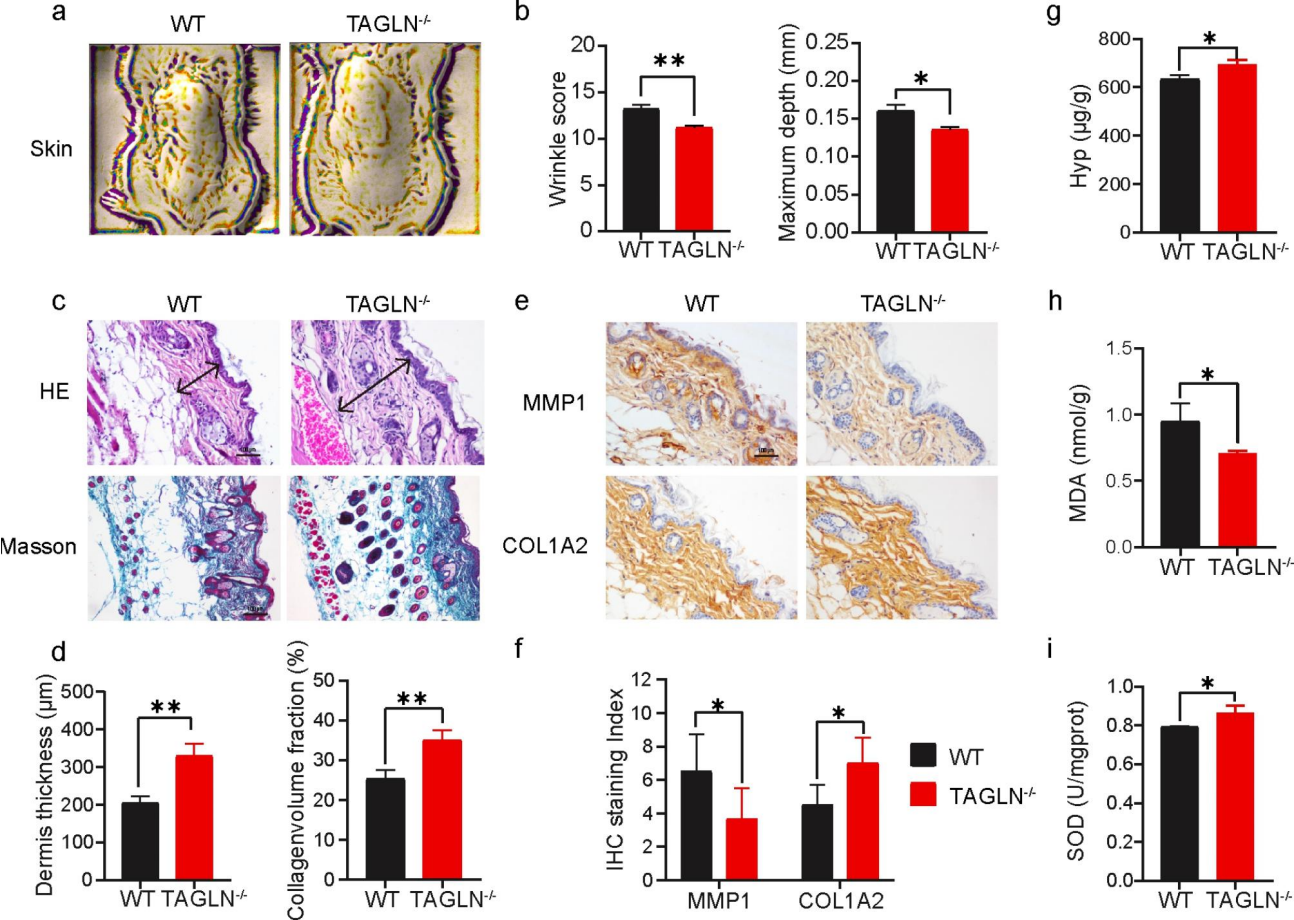
Aging phenotype of *Tagln*^*−/−*^ mice is alleviated compared with that of WT mice. (**a**) Representative images of the skin in WT mice (left) and *Tagln*^*−/−*^ mice (right) obtained by Antera 3D. (**b**) Wrinkle score and maximum depth measured by Antera 3D. (**c** and **d**) H&E staining showing the dermis thickness of *Tagln*^*−/−*^ mice skin and Masson staining showing the accumulation of collagen (blue). Scale bar: 100 μm. e and f) IHC analyses of MMP1 and COL1A2 in the skin of WT and *Tagln*^*−/−*^ mice. Representative images (**e**) and staining scores (**f**) are shown. Scale bar: 100 μm. (**g** and **i**) Levels of Hyp (**g**), MDA (**h**), and SOD (**i**) in WT and *Tagln*^*−/−*^ mice. All values are expressed as mean ± SD, n.s, not significant, ^*^P < 0.05, ^**^P < 0.01

During daily mouse husbandry, we noticed that 20-month-old *Tagln*^*−/−*^ mice had fewer dorsal skin folds than wild-type mice of the same age. We conducted a more accurate and objective analysis through Atera 3D, and the results showed that the skin wrinkle score and wrinkle depth of *Tagln*^*−/−*^ mice were lower than those of WT mice (Fig. 1a and b). Skin aging is often accompanied by the loss of collagen fibers and the thinning of the dermis. Hematoxylin–eosin (H&E) staining showed that the dermis of WT mice was 205.6 ± 18.33 μm thick, whereas that of *Tagln*^*−/−*^ mice was 330.0 ± 32.81 μm thick. Masson staining results indicated that WT mice also exhibited more collagen fiber loss than *Tagln*^*−/−*^ mice (Fig. 1c–d). The loss of type I procollagen in the skin is dependent on the expression of matrix metalloproteinase 1 (MMP1), which is a zinc-dependent endopeptidase that specifically degrades type I collagen. Immunohistochemistry (IHC) was performed to test the expression of MMP1 and COL1A2 in WT and *Tagln*^*−/−*^ mice skin (Fig. 1e). IHC scores were calculated based on the combination of staining area and staining intensity. The results confirmed that compared with those in WT mice, collagen I expression was higher and MMP1 expression was lower in the skin of *Tagln*^*−/−*^ mice (Fig. 1f). Hydroxyproline (Hyp) is one of the specific amino acids of collagen. Hyp content can reflect the change in collagen content in the dermis and indicate the degree of skin aging. The results revealed that the Hyp content of the WT mice skin was 632.9 ± 18.31 µg/g. The Hyp content in the *Tagln*^*−/−*^ mice skin was 693.8 ± 17.70 µg/g, which was higher than that in WT mice (Fig. 1g). The malondialdehyde (MDA) level in the skin is often considered an index for evaluating the peroxidation of lipid metabolites. The MDA level in WT mice’s skin was 0.948 ± 0.138 nmol/g, which was higher than that in the skin of *Tagln*^*−/−*^ mice (0.711 ± 0.018 nmol/g, Fig. 1h). Superoxide dismutase (SOD) is a free radical scavenger that plays an important role in the skin’s antioxidant defense system. SOD activity in the skin of WT and *Tagln*^*−/−*^ mice was 0.7929 ± 0.005 and 0.8641 ± 0.039 U/mg prot, respectively (Fig. 1i). The results illustrated that the development of aging-associated skin phenotypes in *Tagln*^*−/−*^ mice were delayed relative to that in WT mice.

### TAGLN and ZEB1 expression is related to UVA-induced cell senescence

**Fig. 2:**
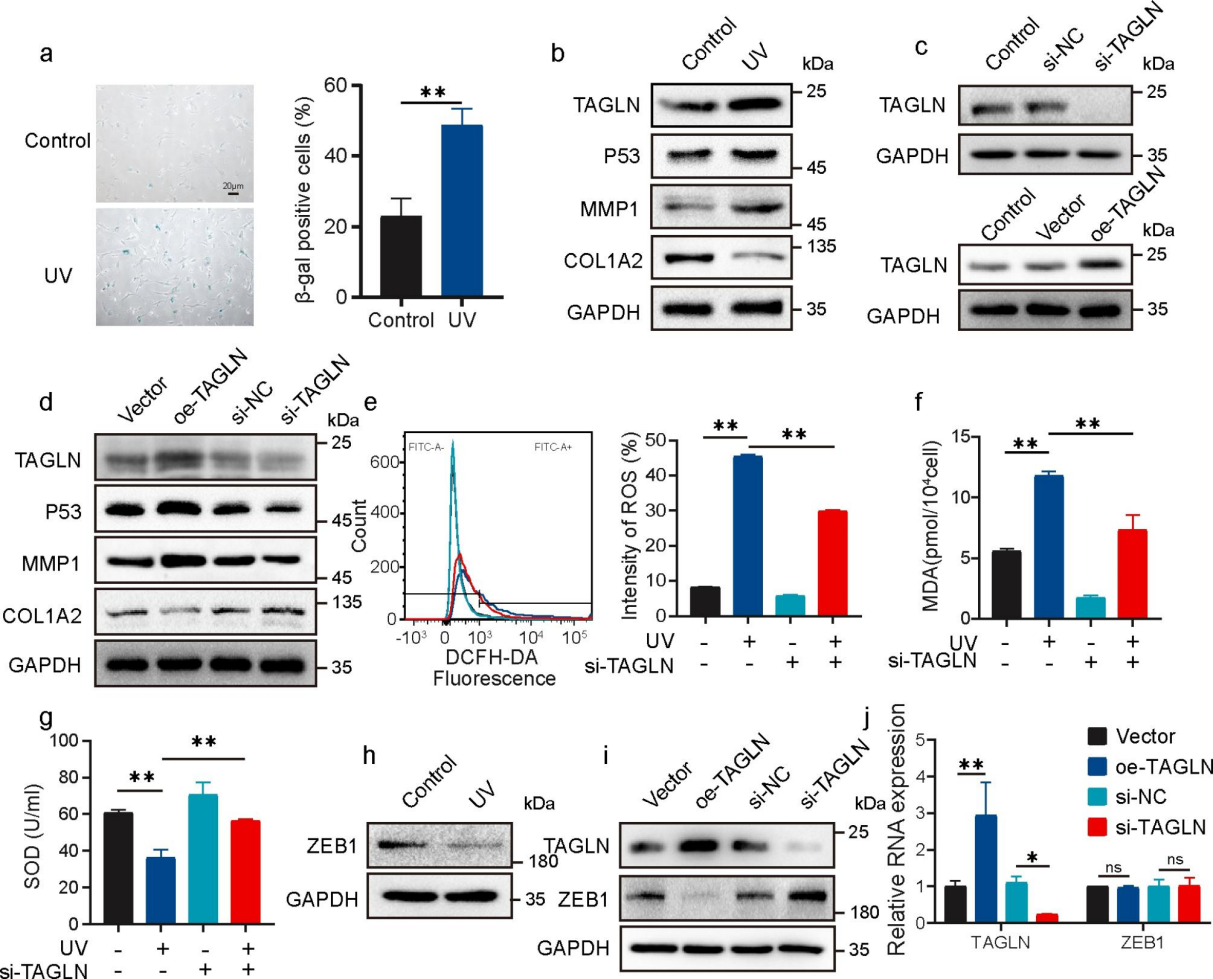
TAGLN and ZEB1 expression levels are associated with UVA-induced cellular senescence in HSFs. (**a**) SA-β-Gal staining assay in UV-induced HSFs. Scale bar: 20 μm. (**b**) Expression level changes of TAGLN, P53, MMP1, and COL1A2 after UVA irradiation as detected by Western blot. (**c**) Western blot images showing TAGLN expression level after TAGLN overexpression or knockdown. (**d**) P53, MMP1, and COL1A2 protein expression level in HSFs after TAGLN overexpression or knockdown as determined via Western blot. (**e**) Effect of TAGLN on the ROS content of UV-treated cells. Typical flow cytometry profiles (left) and quantitative analysis (right) of the fluorescence intensity of FITC (ROS) in HSFs. (**f** and **g**) MDA concentrations (**f**) and total SOD activities (**g**) in HSFs treated with TAGLN siRNA or UVA irradiation. MDA, malondialdehyde; SOD, superoxide dismutase. (**h**) ZEB1 expression level change in HSFs after UVA irradiation. (**i** and **j**) TAGLN and ZEB1 expression in HSFs at the mRNA (**j**) and protein (**i**) levels following TAGLN overexpression or knockdown as determined via qPCR and Western blot analyses, respectively. All values are expressed as mean ± SD, n.s, not significant, ^*^P < 0.05, ^**^P < 0.01

UVA was used to irradiate HSFs to establish a cell aging model to explore the effect of TAGLN on skin aging. Senescence-associated β-galactosidase (SA-β-Gal) staining was performed to analyze the degree of cell senescence. The β-galactosidase staining rate of UVA-stressed HSFs was 48.67%±4.64%, whereas that in control cells was 22.87% ±5.11% (Fig. 2a). The activation of MMP-1 and the degradation of type I collagen are two main markers related to skin aging. Western blot analysis revealed that COL1A2 expression level decreased whereas MMP1 expression level increased in HSFs after UVA irradiation. Consistent with this result, the expression levels of senescence marker P53 and TAGLN remarkably increased after UVA irradiation (Fig. 2b). These data showed that UVA-induced the high TAGLN expression level and the aging state in HSFs. The overexpression vector of TAGLN and the corresponding siRNA were constructed, and efficiency validation was carried out (Fig. 2c) to further explore the role of TAGLN in skin aging. The overexpression or knockdown of TAGLN revealed that TAGLN had a regulatory effect on the expression levels of the aging-related markers, P53, MMP1, and COL1A2, and that its regulatory effect on P53 was the most remarkable (Fig. 2d). The intracellular ROS and MDA levels in HSFs markedly increased by 5.54-fold and 2.12-fold, respectively, after UVA irradiation (Fig. 2e–f). Chronic exposure to UV irradiation caused a remarkable reduction in SOD levels. The SOD level in the UVA irradiation group was 59.4% of that in the control group (Fig. 2g). TAGLN downregulation partially reversed the UVA-induced decrease in SOD and the upregulation of MDA and ROS (Fig. 2e–g). Intracellular ROS have been reported to be responsible for the regulation of ZEB1 expression [16]. Moreover, the expression and function of P53 can be affected by ZEB1 [1, 17]. Western blot analysis confirmed that ZEB1 expression decreased after UVA induction (Fig. 2h). Interestingly, TAGLN overexpression or knockdown can negatively regulate the protein expression level but hardly affected the mRNA level of ZEB1 (Fig. 2i–j). The results suggested that TAGLN can indirectly regulate ZEB1 expression in UVA-induced cell senescence.

### TAGLN reduces the interaction between ZEB1 and USP1 by retaining USP1 in the cytoplasm

**Fig. 3:**
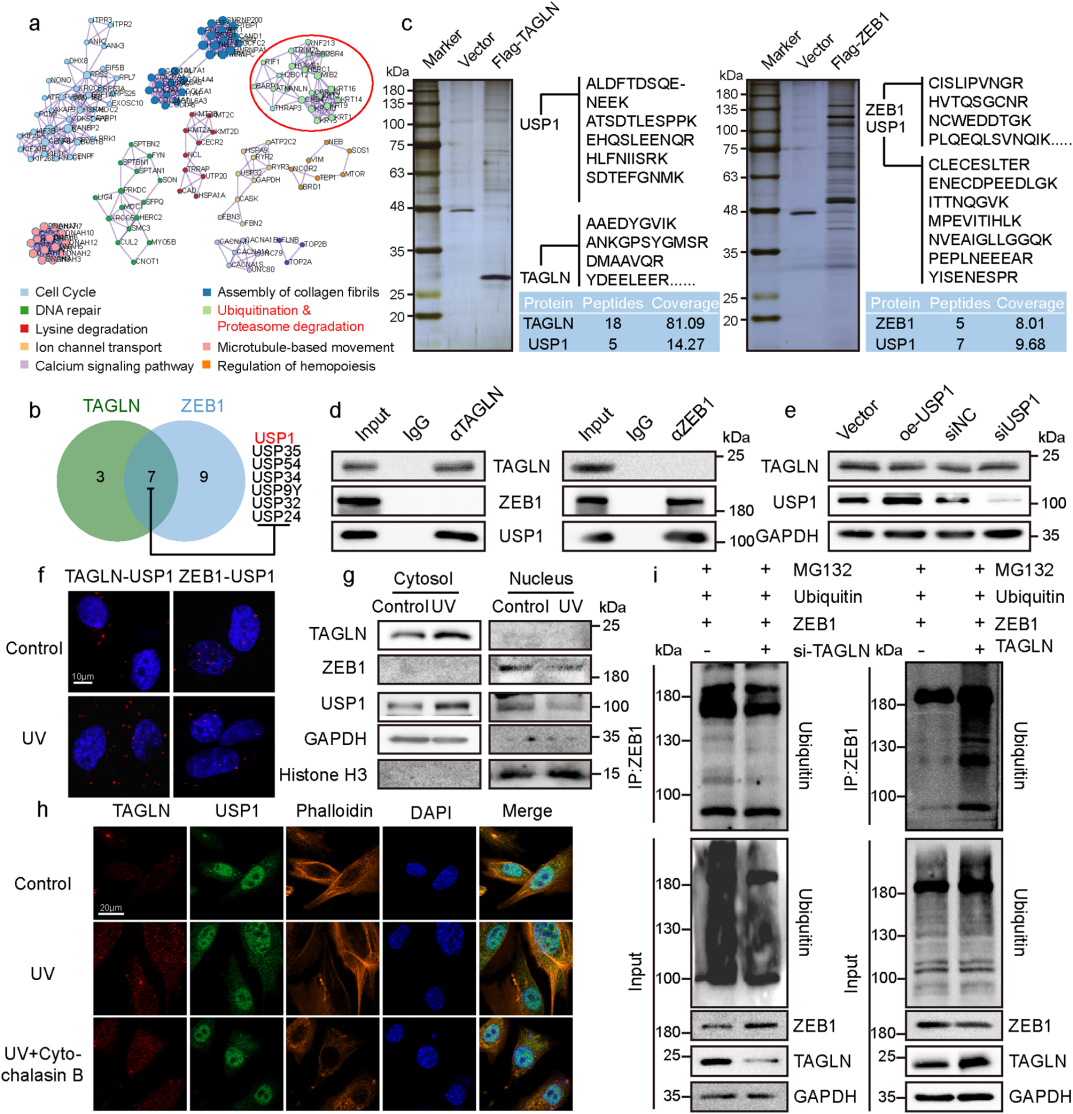
TAGLN reduces the interaction between ZEB1 and USP1 by competitive interaction with USP1. (**a**) PPI enrichment map of the intersection of the respective interacting proteins of TAGLN and ZEB1. (**b**) Venn diagram of the DUBs interacting with TAGLN or ZEB1. (**c**) Pull-down and mass spectrometry analyses of TAGLN- (left) and ZEB1-associated proteins (right). (**d**) Whole-cell lysates from HSFs immunoprecipitated and immunoblotted with antibodies against the indicated proteins. (**e**) TAGLN and USP1 expression levels in HSFs following USP1 overexpression or knockdown as determined by Western blot. (**f**) Interaction of ZEB1 or TAGLN with USP1 in UVA-induced HSFs as detected via Duolink PLA. (**g**) Cytosolic and nuclear proteins extracted from HSFs after 10 J/cm^2^ UV irradiation and subjected to the Western blot analyses of TAGLN, ZEB1, and USP1. (**h**) IF showing the locations of TAGLN and USP1 in HSFs treated with UVA or UVA + cytochalasin B. (**i**) Effects of TAGLN expression level on ZEB1 ubiquitination levels as shown by Western blot. All values are expressed as mean ± SD, n.s, not significant, ^*^P < 0.05, ^**^P < 0.01

After proving the regulatory effect of TAGLN on ZEB1 protein expression, we further investigated the molecular mechanism by which TAGLN regulates ZEB1. TAGLN and ZEB1 have different cell localizations; therefore, this regulation may be mediated by a protein interacting with them. First, the interaction proteins of TAGLN and ZEB1 were extracted through the pull-down assay. After mass spectrometry (MS) analysis, protein–protein interaction (PPI) enrichment analysis was performed on the overlapping proteins that interacted with TAGLN and ZEB1. In the enrichment results, ubiquitination and proteasome degradation are important protein degradation regulation processes in cells, which are closely related to protein expression levels (Fig. 3a). Accordingly, the interaction proteins of TAGLN and ZEB1 included lots of USP proteins, which constitute the largest family of DUB, and the abundance of USP1 was the highest in the intersection of proteins having interaction with TAGLN and ZEB1 (Fig. 3b–c). Co-immunoprecipitation (Co-IP) experiments further confirmed the absence of a direct interaction between TAGLN and ZEB1. USP1 may mediate the regulatory effect of TAGLN on ZEB1 expression level (Fig. 3d). Western blot analysis was conducted to research the functional importance of the interaction between TAGLN and USP1. The results showed that TAGLN expression did not remarkably change after USP1 overexpression or knockdown (Fig. 3e). Consistently, TAGLN also did not affect the USP1 expression level (Fig. S1a). The Duolink experiment revealed that after UVA irradiation, the signal dots of the interaction between TAGLN and USP1 increased remarkably and were specifically located in the cytoplasm. Whereas the interaction signals between ZEB1 and USP1 decreased substantially after UVA irradiation (Fig. 3f). When we knocked down TAGLN, the interaction signal between ZEB1 and USP1 increased (Fig. S1b). As inferred from the above data, TAGLN competed with ZEB1 for binding with USP1. Nucleoprotein and cytoplasmic proteins were extracted for Western blot analysis. The results illustrated that USP1 increased in the cytoplasm and decreased in the nucleus after UV irradiation, and the ZEB1 expression level in the nucleus decreased synchronously with that of USP1 (Fig. 3g and S1c). Previous studies indicated that TAGLN interacts with actin, and we speculated that the colocalization of TAGLN and the cytoskeletal protein actin may contribute to the retention of USP1 in the cytoplasm. Immunofluorescence (IF) results demonstrated that TAGLN and USP1 were found to co-localize with microfilaments when actin filaments were visualized with phalloidin. The colocalization signals of TAGLN and USP1 on microfilaments increased after UV irradiation but became discrete after the structure of the microfilaments was destroyed by cytochalasin B (Fig. 3h). The ubiquitination assay experiment demonstrated that ZEB1-bound ubiquitin increased remarkably after TAGLN overexpression but decreased accordingly after TAGLN knockdown (Fig. 3i). Western blot results showed that ZEB1 expression decreased when TAGLN and USP1 were knocked down at once (Fig. S1d). All the results indicated that TAGLN retains USP1 in the cytoplasm and regulates the stability of ZEB1 via regulating its ubiquitination level.

### USP1 protects ZEB1 from ubiquitination degradation

**Fig. 4:**
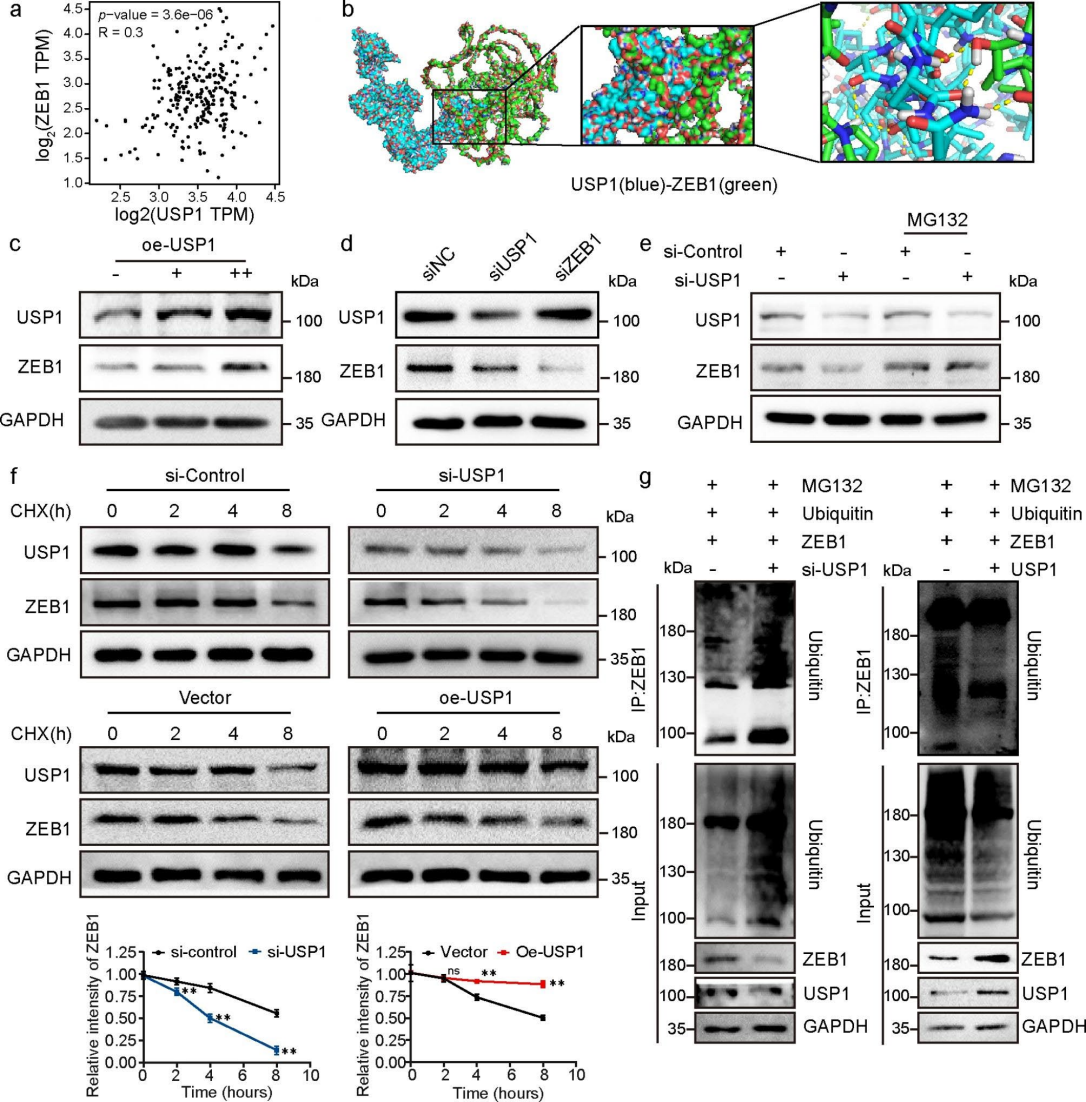
USP1 stabilizes ZEB1 by deubiquitination. (**a**) Statistical analysis of the correlation between USP1 and ZEB1 expression in human skin samples from the GEPIA database (P < 0.05, R = 0.3). (**b**) Detailed binding mode of USP1 (blue) and ZEB1 (green). (**c**) Effect of USP1 overexpression on ZEB1 expression level as detected by Western blot. (**d**) Effect of USP1 or ZEB1 knockdown on each other expression level as analyzed by Western Blot. (**e**) HSFs were transfected with control siRNA or USP1 siRNA with or without 50 µM MG132 and then subjected to Western blot analysis using anti-USP1 and ZEB1 antibody. (**f**) Cycloheximide chasing experiment showing ZEB1 stability after USP1 overexpression or knockdown. (**g**) Effects of USP1 on ZEB1 ubiquitination levels. All values are expressed as mean ± SD, n.s, not significant, ^*^P < 0.05, ^**^P < 0.01

The PPI interface after the virtual docking between ZEB1 and USP1 is shown in Fig. 4b. Considering that USP1 functions as a DUB, we further verified whether USP1 can stabilize ZEB1 via deubiquitination. The correlation analysis based on the Gene Expression Profiling Interactive Analysis (GEPIA) database revealed a positive correlation between the expression levels of ZEB1 and USP1 in the skin (Fig. 4a). Western blot analysis demonstrated that ZEB1 expression level increased after USP1 overexpression and decreased after USP1 knockdown. However, ZEB1 knockdown had a negligible effect on USP1 expression (Fig. 4c–d). Proteasomes play a key role in ubiquitination degradation. The reduction in ZEB1 protein level due to USP1 depletion could be rescued after the addition of the proteasome inhibitor, MG132 (Fig. 4e). These data supported the conclusion that USP1 can stabilize ZEB1 via deubiquitination. For confirmation, cycloheximide (CHX) chase assays were conducted to detect the dynamic changes in ZEB1 under USP1 knockdown or overexpression treatments. The results showed that the half-life of ZEB1 shortened from 8 h to approximately 4 h after USP1 knockdown. Conversely, compared with that under the control treatment, the half-life period of ZEB1 became substantially longer after USP1 overexpression (Fig. 4f). Furthermore, the deubiquitination assays confirmed that the level of ZEB1-bound ubiquitin was remarkably increased in USP1-knocked down cells and reduced when USP1 was overexpressing, ZEB1-bound ubiquitin level was restored after USP1-specific inhibitor SJB3-019 A intervention (Fig. 4g and S2). The results supported the conclusion that USP1 protects ZEB1 through deubiquitination.

### Zer can target the TAGLN/USP1 interactive interface and promotes the binding between USP1 and ZEB1

**Fig. 5:**
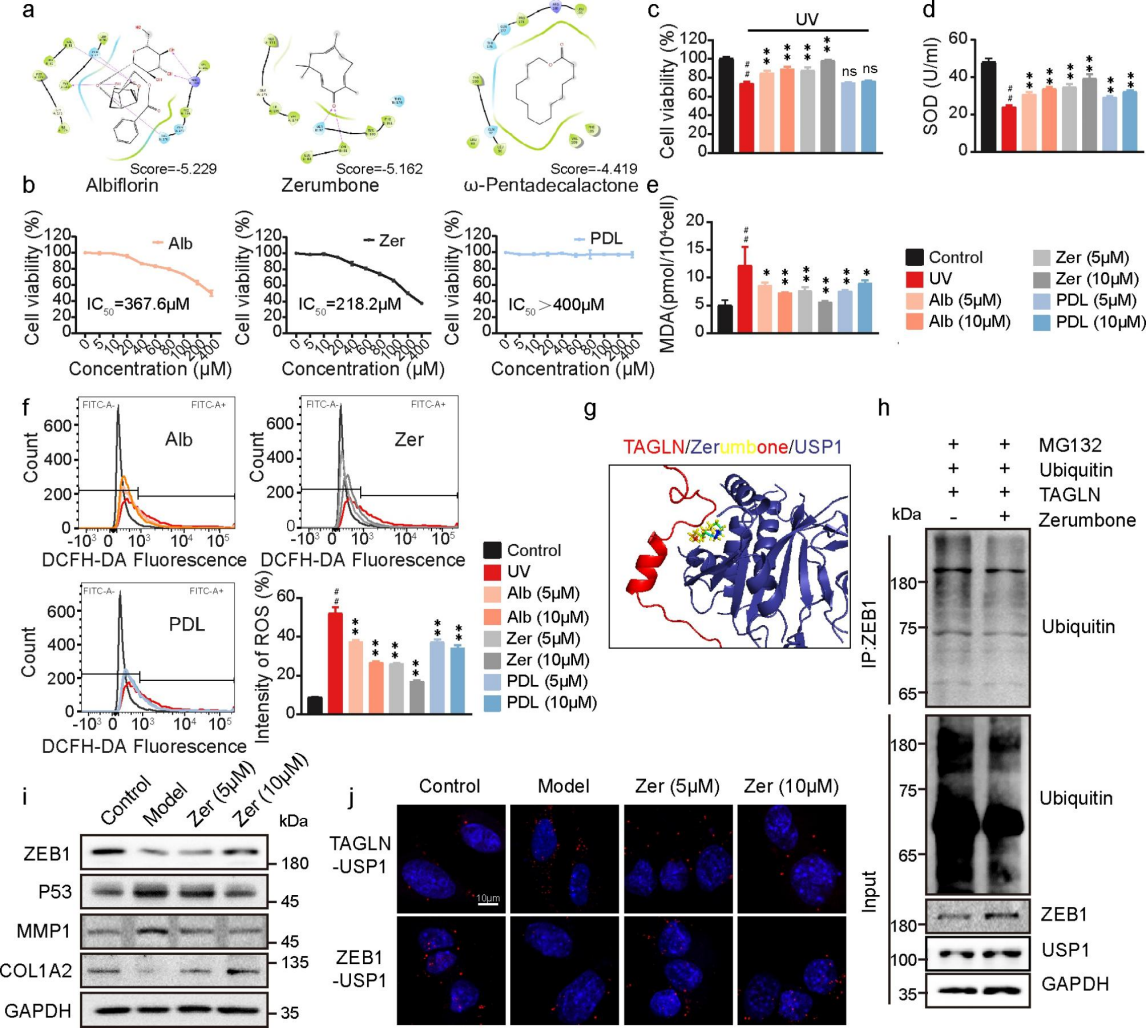
Zer targets the TAGLN/USP1 interface and promotes USP1 binding to ZEB1. (**a**) 2D diagram of the ligand interaction of Alb, Zer, or PDL with TAGLN. (**b**) Effects of Alb, Zer, and PDL on cell viability detected by CCK-8 assay. (**c**) Effects of Alb, Zer, and PDL on UV-induced cell damage examined by CCK-8 assay. (**d**–**f**) SOD (**d**), MDA (**e**), and ROS (**f**) levels of HSFs under Alb, Zer, or PDL pre-protection before UVA irradiation. g) Predicted binding modes of Zer and the TAGLN/USP1 complex. **h**) Effect of Zer on ZEB1 ubiquitination levels. **i**) Western blot analyses of ZEB1, P53, MMP1, and COL1A2 in HSFs treated with UVA and different Zer concentrations. **j**) Interaction of ZEB1 or TAGLN with USP1 in HSFs treated with 5 or 10 µM Zer as detected by Duolink PLA. All values are expressed as mean ± SD, n.s, not significant, ^*^P < 0.05, ^**^P < 0.01 compared with the UV-treated group. ^#^P < 0.05, ^##^P < 0.01 compared with the control group

TAGLN plays an indispensable role as a cytoskeletal protein. We proposed a strategy to enhance the stability of ZEB1 by inhibiting the interaction between TAGLN and USP1 and reducing the retention of USP1 in the cytoplasm. According to previous studies, some macrocyclic monomers have excellent binding activity to TAGLN [18]. Therefore, we randomly selected 200 plant-derived extracts with macrocyclic monomer and screened for inhibitors of the TAGLN/USP1 interactive interface by virtual docking. Leading compounds with high scores, including albiflorin (Alb, − 5.229), Zer (− 5.162), and ω-pentadecalactone (PDL, − 4.419), were screened out. The docking conformation is shown in Fig. 5a. First, the safety of the three leading compounds was determined through the cell counting kit-8 (CCK-8) experiment. The results showed that the IC_50_ of Alb, Zer, and PDL are 367.6, 218.2, and > 400 µM, respectively, which means that the three leading compounds had low toxicity to HSFs (Fig. 5b). In further experiments, we tested the effects of the three leading compounds at different doses (5 and 10 µM) on UVA-induced cell viability. The cell viability in the UV irradiation group was 74.05%±1.525%. Compared with those in the UV irradiation group, the cell activities increased by 10.56% and 13.9% in the 5 µM Alb and Zer pre-protection groups, respectively, and by 15.41% and 24.23% in the 10 µM Alb and Zer pre-protection groups, respectively. Although PDL had the lowest cytotoxicity, its activity against UVA irradiation was not ideal. The cell viability in the 10 µM PDL pre-protection group was not substantially different from that in the UVA irradiation group (Fig. 5c). The levels of SOD, MDA, and ROS, the three important indexes of cell aging, were tested at different drug dosages to explore the effect of the three leading compounds on UVA-induced cell aging. The results indicated that compared with that in the control group, the SOD activity in the UVA irradiation group decreased by 49.73% and was restored to 82.12% in the 10 µM Zer pretreatment group, which showed the best protection effect (Fig. 5d). Compared with those in the control group, MDA increased by 2.4-fold and the positive rate of ROS increased by 43.27% in the UVA irradiation group. Among the three leading compounds, Zer had the most remarkable inhibitory effect on MDA and ROS accumulation (Fig. 5e–f). Therefore, we selected Zer for further study. Zer acts as a TAGLN/USP1 interactive interface inhibitor, and the action site is shown in Fig. 5g. The deubiquitination experiment results showed that the level of ZEB1-bound ubiquitin decreased markedly after Zer treatment (Fig. 5h). As the results of Western Blot showed, compared with those in the UVA irradiation group, ZEB1 expression level increased in a dose-dependent manner, and the expression levels of P53 and MMP1 decreased accordingly, further increasing the expression level of COL1A2 in the Zer pre-protection group (Fig. 5i). The detection results of the mRNA level of the downstream target gene of ZEB1 also supported the anti-aging effect of Zer. Among them, the mRNA levels of TIMP2 related to collagen level and NFKBIA related to inflammation suppression decreased with the decrease of ZEB1 expression level after UV irradiation. However, after Zer intervention, the mRNA levels of TIMP2 and NFKBIA were restored (Fig. S3a). The inhibitory effect of Zer on TAGLN/USP1 interaction was verified via a Duolink experiment. The results showed that compared with the model group, the retention of USP1 by TAGLN decreased after Zer treatment, and the corresponding binding of USP1 to ZEB1 was increased (Fig. 5j). To further confirm the inhibitory effect of Zer on the interaction between TAGLN and USP1, we performed a GST pull-down assay. The results showed that Zer could dose-dependently inhibit the interaction between TAGLN and USP1 in vitro (Fig. S3b). The above results illustrated that Zer competitively binds with TAGLN to reduce the retention of USP1 in the cytoplasm and therefore indirectly plays a role in protecting ZEB1.

### Zer inhibits UVA-induced skin aging

**Fig. 6:**
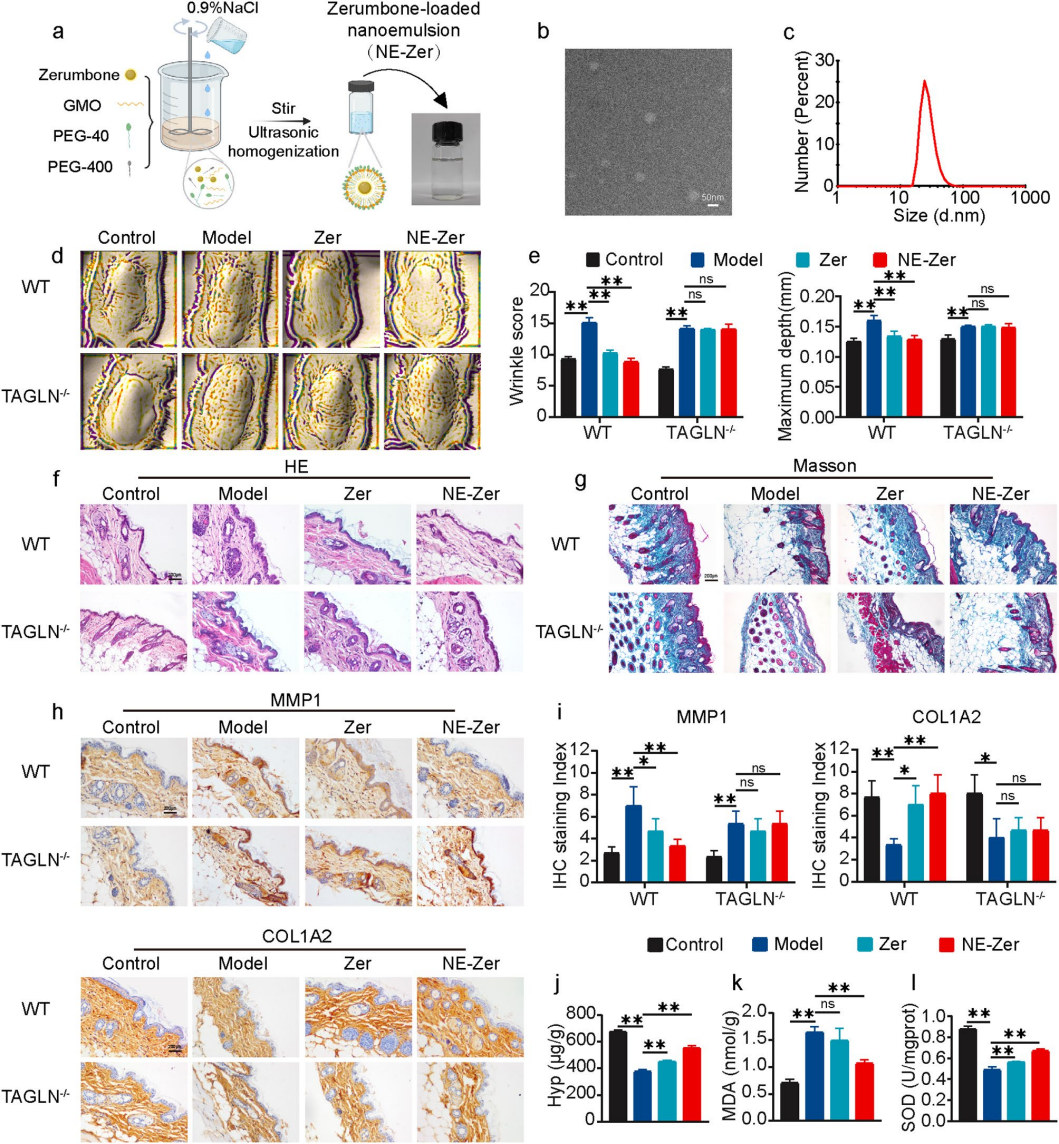
Zer-loaded nanoemulsion inhibits UVA-induced skin aging. (**a**) Schematic of the preparation of Zer-loaded nanoemulsion (NE-Zer). (**b**) Representative TEM image of the Zer-loaded nanoemulsion. (**c**) Particle size distribution of the Zer-loaded nanoemulsion as determined via DLS. (**d** and **e**) Antera 3D plots of WT and *Tagln*^*−/−*^ mice treated with Zer or NE-Zer. The wrinkle score and maximum depth are shown in the right panel. (**f**) Representative H&E-stained skin sections in each group of WT and *Tagln*^*−/−*^ mice with Zer or NE-Zer treatment before UV irradiation. (**g**) Masson staining of collagen (blue) in the skin sections of WT and *Tagln*^*−/−*^ mice with Zer or NE-Zer treatment before UV irradiation. (**h** and **i**) IHC analyses of MMP1 and COL1A2 in the skin sections of WT or *Tagln*^*−/−*^ mice with Zer or NE-Zer treatment before UV irradiation (**h**). Scale bar: 200 μm. Representative images and staining scores are shown (**i**). (**j**–**l**) Hyp (**j**), MDA (**k**), and SOD (**l**) levels in WT and *Tagln*^*−/−*^ mice with Zer or NE-Zer treatment before UV irradiation. All values are expressed as mean ± SD, n.s, not significant, ^*^P < 0.05, ^**^P < 0.01

The potential of Zer as an antiaging active small molecule was further evaluated using UVA-induced skin aging mouse models. However, the barrier function of the skin and the damaging thickening of the epidermis often lead to poor permeability of active substances and unsatisfactory effects. Therefore, as shown in Fig. 6a, a nanoemulsion rich in Zer (NE-Zer) was prepared to improve the bioavailability and therapeutic effect of Zer. NE-Zer was characterized using transmission electron microscopy (TEM) and dynamic light scattering (DLS). The results illustrated that nanoemulsion loaded with Zer had a particle size of 47.75 ± 21.23 nm and uniform dispersion (Fig. 6b–c). In WT mice, NE-Zer applied once a day was more effective in preventing wrinkle formation than Zer (Fig. 6d), and the wrinkle score and maximum depth showed substantial reductions (Fig. 6e). The administration of Zer or NE-Zer had no remarkable effect in *Tagln*^*−/−*^ mice, which is likely due to target deletion. Animal models of aging established by UVA irradiation first exhibit thicken of the epidermis, which further leads to loss of collagen and thinning of the dermis. H&E and Masson staining results demonstrated that NE-Zer can prevent the stress-induced thickening of the epidermal layer and the loss of collagen in the skin in WT mice (Fig. 6f–g). Changes in ZEB1 expression levels detected in the skin tissues were consistent with those in cells, which decreased after UV irradiation. NE-Zer treatments could protect ZEB1 better but did not affect the expression levels of USP1 (Fig. S4). The inhibitory effect of NE-Zer on MMP1 expression was also reflected by the IHC results. Accordingly, COL1A2 expression increased in the NE-Zer-treated group compared with the UVA radiation group (Fig. 6h–i). On the basis of comprehensive considerations, NE-Zer showed more obvious antiaging activity in the aging-induced WT mouse model than in the *Tagln*^*−/−*^ mouse model. The levels of the typical aging markers, Hyp, MDA, and SOD in the skin, were further examined. Compared with those in the aging-induced model group, Hyp and SOD increased by 1.20-fold and 1.16-fold, respectively, whereas MDA decreased by 8.95% in the Zer-treated group. The antiaging pre-protection effect of Zer on the skin remarkably improved after Zer was prepared into NE-Zer. Hyp and SOD increased by 1.46-fold and 1.38-fold, respectively, whereas MDA decreased by 35.06% under the protection of NE-Zer compared with those in the model group (Fig. 6j–i). The above results verified that Zer targets TAGLN and can play a protective role against aging in mice models. After being prepared into a Zer-rich nanoemulsion, the anti-aging protection effect is significantly improved.

## Discussion

UV irradiation can lead to numerous human skin diseases [19]. Long-term exposure to UV rays causes the senescence-associated secretory phenotype (SASP) [20]. TAGLN is an actin-related protein, which is related to cell proliferation, migration, and invasion [21, 22]. Studies on TAGLN found that its imbalance can promote the development of rectal, breast, and colorectal cancers, and is considered to be a tumor suppressor [23–26]. Existing studies have shown that the mRNA level of TAGLN is increased in the UV-induced premature aging model of human skin fibroblasts [12]. In the present work, we found that the skin aging degree of *Tagln*^*−/−*^ mice is remarkably lower than that of WT mice. In addition, the expression level of TAGLN increased after UV treatment. The above phenomena show that TAGLN may play a regulatory role in UV-induced skin aging.

ZEB1 expression level is often inhibited by ROS, which can be induced by UVA irradiation. ZEB1 upregulation could partially reverse the UVA-induced increase in SA-β-gal-positive cells and the upregulation of p53, p21, and p16 expression levels, whereas the ZEB1 downregulation exerts the opposite effect. Therefore, ZEB1 plays a key role in UVA-induced aging [16, 27]. This work demonstrated that the ZEB1 expression level was downregulated after UVA irradiation, and the expression of downstream aging-related markers, including MMP1 and P53, increased.

In this study, the molecular mechanism underlying the regulatory effect of TAGLN on ZEB1 expression level was deeply explored. The results indicated that TAGLN overexpression or knockdown can regulate the protein expression level but not the mRNA level of ZEB1. The results suggested that TAGLN may indirectly regulate ZEB1 expression levels in UVA-induced cell aging. The interacting proteins of TAGLN and ZEB1 were identified by MS, and the enrichment analysis of the intersection of the two was carried out. The enrichment results showed that the interaction proteins of TAGLN and ZEB1 included a large number of DUBs and that USP1 had the highest abundance. Existing studies have demonstrated that the decrement in ZEB1 depends on ubiquitination degradation, and deubiquitinating enzymes such as USP51, USP7 and USP17 can all act on ZEB1. We further explored whether USP1 affected the ubiquitination level of ZEB1. The Duolink experimental results showed that the interactive signal points between TAGLN and USP1 remarkably increased after UV irradiation and were specifically located in the cytoplasmic. Moreover, the interactive signals between ZEB1 and USP1 decreased. Western blot results showed that the expression level of ZEB1 also increased or decreased after overexpression or knockdown of USP1 while changing ZEB1 hardly affected the expression level of USP1. Based on the above results, USP1 can affect the expression level of ZEB1 by regulating the ubiquitination level of ZEB1. Moreover, TAGLN can competitively inhibit the binding between USP1 and ZEB1, that is, TAGLN retained USP1 in the cytoplasm and regulates ZEB1 expression level via the ubiquitination pathway. This study demonstrated that, in addition to binding to actin to affect cell migration and invasion, TAGLN can regulate the aging of skin cells by regulating the ubiquitination level of ZEB1.

Zer is an active compound isolated from the wild ginger plant, *Zingiber zerumbet* (L.) [28]. Current studies on Zer focused on its anticancer activity [29]. In addition, Zer executes various biological activities, including antihyperlipidemic, antidiabetes, antiatherosclerosis, and antioxidative effects [30–32]. In this work, Zer was screened as an interactive interface inhibitor that competitively inhibits the interaction between USP1 and TAGLN to promote the binding between USP1 and ZEB1 and thus protect ZEB1 from ubiquitination degradation. Zer pretreatment had a remarkable inhibitory effect on ROS and MDA accumulation. Moreover, Zer had a protective effect against the damage of UVA to SOD activity. Therefore, Zer may be the leading compound with the most potential for preventing skin aging. Given the barrier effect of the skin itself and the thickening of the damaged epidermal layer, the liposoluble Zer has poor permeability. In this work, Zer was prepared as a nanoemulsion to improve its bioavailability by enhancing its transdermal absorption efficiency.

In conclusion, we found that TAGLN can competitively inhibit the binding between USP1 and ZEB1 by interacting with USP1 and can promote ZEB1 degradation to regulate UVA-induced photoaging. Zer, as an interactive interface inhibitor of TAGLN/USP1, can inhibit ZEB1 degradation and therefore inhibit UVA-induced photoaging.

## Materials and methods

### Cell culture and transfection

HSFs were purchased from Cellcook (Guangzhou, China). The cells were maintained in Dulbecco’s modified Eagle medium (Bioind, ISR) with 10% fetal bovine serum (Bioind, ISR) and 1% penicillin/streptomycin (Hyclone, USA) in a humidified incubator at 37 °C with 5% CO_2_. All the plasmids or siRNAs were transfected into cells using Lipofectamine 8000 (Beyotime Biotechnology, China).

### Cellular senescence model and SA-β-Gal staining

The cell senescence model was constructed by replacing the complete medium with a small volume of PBS. The cells were exposed to 10 J/cm^2^ UVA irradiation for 20 min, transferred into a humidified incubator at 37 °C with 5% CO_2_, and cultured for 6 h. β-Galactosidase staining was performed according to the manufacturer’s guidelines. Cells were fixed at room temperature for 15 min, washed three times with PBS, added staining working solution, incubated overnight at 37 ℃, and observed by optical microscope.

### UVA-induced skin aging model in mice

C57BL/6 mice (female, 6–8 weeks) were kept in a specific pathogen-free animal care facility. The mice were allowed to acclimate for 7 days before the experiment. All animal studies were conducted in accordance with the animal use guidelines of the National Institutes of Health and China’s current regulations and standards on the use of laboratory animals. All animal procedures were approved by the Animal Ethics Committee of Tianjin International Joint Academy of Biomedicine. For the establishment of the UVA-induced skin aging mouse model, a single 1.5 cm×1.5 cm area on the back of a mouse was exposed to UVA radiation. The total UVA dose per irradiation was 5 J/cm^2^. The experimental animals were exposed to UVA radiation three times a week for two consecutive weeks. Antera® 3D was used to photograph the back of the mouse skin. The images collected daily were unaffected by the surrounding light conditions.

### Western blot analysis

The cells were washed twice with ice-cold PBS and ruptured on ice with radioimmunoprecipitation (RIPA) assay buffer (KeyGEN BioTECH, Nanjing, China), phenylmethylsulfonyl fluoride (PMSF), and an inhibitor cocktail for 30 min. The mixture was centrifuged at 12 000×*g* for 15 min at 4 °C, and the supernatant was collected. Protein concentration in the lysates was determined using a bicinchoninic acid (BCA) protein assay kit (ThermoFisher Scientific, USA) in accordance with the manufacturer’s protocol. The sample was added with bromophenol blue-containing loading buffer, boiled for 10 min, resolved through 10% SDS–polyacrylamide gel electrophoresis (SDS-PAGE), and finally transferred to a polyvinylidene fluoride (PVDF) membrane (Millipore, USA). After blocking with 5% milk, the membrane was incubated with appropriate primary antibodies at 4 °C overnight, and then incubated with secondary antibodies. The primary antibodies included COL1A2 (1:1000, Affinity), MMP1 (1:1000, Affinity), p53 (1:1000, Affinity), TAGLN (1:1000, Proteintech), ZEB1 (1:1000, Proteintech), USP1 (1:1000, Proteintech), and GAPDH (1:5000, Affinity). Detection was performed using an enhanced chemiluminescence kit (Vazyme, Nanjing, China).

### Histological analysis of skin tissues

Mouse skin tissues fixed in 10% paraformaldehyde for 48 h and embedded in paraffin were sliced into 5 μm-thick sections. For H&E staining, the paraffin-embedded skin tissue sections were deparaffinized in xylene, rehydrated with ethanol at a reduced concentration, and stained using an H&E detection kit (Solarbio, China) and modified Masson’s trichrome stain kit (Solarbio, China) following the manufacturer’s instructions. Histological observation was then performed with an optical microscope.

### Immunohistochemistry

For staining, skin tissue sections were deparaffinized, incubated in xylene and a graded ethanol series, and treated with 3% H_2_O_2_ to block endogenous peroxidase activity. After blocking, the samples were incubated with MMP1 antibody (1:100) or COL1A2 antibody (1:100) at 4 °C overnight. The slides were then washed with PBS twice. A HRP-polymer antimouse/rabbit IHC kit (Maixin Biotech, Fuzhou, China) was used to incubate the skin tissue sections with the secondary antibody at room temperature. Detection was performed using a DAB HRP color development kit (Beyotime Biotechnology, China) in accordance with the manufacturer’s protocol.

### Immunofluorescence

The cells were fixed in 4% paraformaldehyde for 15 min at room temperature and blocked with 5% BSA and 0.03% Triton X-100 for 20 min. After washing with PBS, the cells were incubated with primary antibodies at 4 °C overnight. The cells were incubated with secondary antibodies (Beyotime, China) at 37 °C for 1.5 h, and nuclei were stained with DAPI (Beyotime, China). A confocal laser microscope (Zeiss LSM800) was used for observation.

### RNA extraction and RT-PCR

The total RNA extracted from the cells was reverse transcribed into cDNA by using a PrimeScript RT reagent kit (Tiangen, China). A SYBR RT-PCR kit (Tiangen, China) was used for transcript quantification with specific primers. Expression levels were quantified using the 2^−ΔΔCt^ method with GAPDH as the internal reference.

### Pull down and silver staining

Lysates from HSFs expressing Flag–TAGLN or Flag–ZEB1 were prepared using 0.3% NP-40 lysis buffer containing a protease inhibitor cocktail. Cell extracts were incubated with anti-Flag protein A + G beads (Beyotime, China) for 12 h at 4 °C. After binding, the beads were washed with cold 0.1% NP-40 lysis buffer. The Flag-tagged protein complexes were then eluted with Flag peptide (Sigma, USA). The eluents were collected and visualized on 10% SDS-PAGE and subjected to silver staining with a Fast Silver Stain Kit (Beyotime, China). Distinct protein bands were retrieved and analyzed via MS.

### Molecular docking

Molecular docking was performed using Schrodinger software. The protein crystal structure was downloaded from Protein Data Bank. ClusPro (https://cluspro.bu.edu/dimer_predict/submit.php) was applied to perform protein–protein docking. Small molecules were downloaded from the ChemPub database. The docking score was used to select small molecules.

### ROS measurement

The fluorescent probe (DCFH-DA) was loaded into the cells in situ using a ROS determination kit (Solarbio, China). The cells were incubated with DCFH-DA in a CO_2_ incubator at 37 °C for 20 min. Then, the cells were collected for flow cytometry (Beckton Dickinson, USA).

### Measurement of SOD enzyme activity

A total SOD assay kit (Beyotime, China) was used in accordance with the manufacturer’s instructions to determine SOD enzyme activity in the cells or tissue homogenates. Absorbance was measured at 450 nm with a microplate reader (Thermo Scientific, USA). Then, SOD enzyme activity was calculated.

### Measurement of MDA content

A lipid peroxidation MDA assay kit (Beyotime, China) was used in accordance with the manufacturer’s instructions to determine the MDA content in cells or tissue homogenates quantitatively. The absorbances at 450, 532, and 600 nm were measured using a microplate reader (Thermo Scientific, USA). Then, MDA content was calculated.

### Measurement of HYP content

A HYP content assay kit (Beyotime, China) was used in accordance with the manufacturer’s instructions to determine HYP content in the cells or tissue homogenates. The absorbance at 560 nm was measured using a microplate reader (Thermo Scientific, USA). Then, HYP content was calculated.

### Deubiquitination assay

The cells were lysed with 0.3% NP-40 lysis buffer and centrifuged at 12 000 rpm for 10 min. Then, the cell extracts were incubated with anti-ZEB1 antibody-conjugated protein A + G agarose at 4 °C for 12 h. Subsequently, the samples were washed five times with cold 0.1% NP-40 lysis buffer, boiled in SDS loading buffer, and subjected to SDS-PAGE. The samples were subjected to immunoprecipitation with the indicated antibodies and blotted with anti-Ub antibody.

### Proximity ligation assay (PLA)

PLA was performed in accordance with the manufacturer’s instructions (Thermo Scientific, USA). Briefly, the HSFs were seeded on glass slides and then treated with the above-described experimental conditions. The HSFs were washed, blocked, and incubated with anti-TAGLN (1:200) or anti-ZEB1 (1:200) with anti-USP1 (1:200) primary antibodies at 4 °C overnight. Afterward, the HSFs were washed and incubated with Duolink PLA MINUS and PLUS probes for 1 h at 37 °C. A Duolink in situ detection kit was used for ligation and amplification. DAPI was used to stain the nucleus.

### Preparation and characterization of the nanoemulsion [33]

Zer (60 mg) was added to a 1:8:1:50 mixture of glycerol mono-oleate, PEG-40 hydrogenated castor oil, polyethylene glycol 400 (PEG-400), and normal saline (0.9% NaCl). The ratios refer to the contents of the oil phase, surfactant, cosurfactant, and water phase, respectively. The mixture containing Zer (0.1%, w/v) in addition to the aqueous phase was stirred at 37 °C for 10 min at 300 rpm. Subsequently, an ultrasonic homogenizer (Ningbo Scientz Biotechnology Co., Ltd., Ningbo, China) was used to set the rated power to 99%. Sonication was performed for 5 min to complete mixing. Finally, 0.9% NaCl was added to the mixture obtained in the previous step and stirred gently to obtain a nanoemulsion.

### Physical characterization of the nanoemulsion

Briefly, the nanoemulsion was evaluated through particle size distribution analysis using DLS (Malvern, Nano-ZS, UK). The particle morphology of the nanoemulsion was analyzed through TEM (FEI, Talos F200C, USA). The nanoemulsion (5 µl) was added on a carbon-coated copper grid. The sample was allowed to stand horizontally for 1 min, stained with uranyl acetate, and dried. Talos F200C was used for observation.

### Statistical analysis

Statistical analyses were performed using GraphPad Prism 8 (La Jolla, CA), and the results were expressed as mean ± SD. Differences between groups were assessed through unpaired two-tailed *t* test (for simple two-sample comparison) or one-way ANOVA with Dunnett’s test (for multiple comparisons). Statistical significance was set at ^*^P < 0.05 or ^**^P < 0.01.

## Electronic supplementary material

Below is the link to the electronic supplementary material.

**Additional file 1**: **Fig. S1** a) Western blot analysis of USP1 expression after TAGLN overexpression. b) Duolink PLA demonstrates that USP1 interacts with TAGLN or ZEB1. c) Statistical analysis of Western blot results. d) Western blot analysis of ZEB1 protein expression after the co-knockdown of TAGLN and USP1. **Fig. S2** SJB3-019A inhibited the deubiquitination of ZEB1 by USP1. **Fig. S3** a) TIMP2 and NFKBIA mRNA levels quantified by q-PCR. b) Purified GST-tagged TAGLN was incubated with His-tagged USP1 in the presence or absence of the indicated amounts of Zerumbone. The interaction between TAGLN and USP1 was visualized using immunoblots. **Fig. S4** Western blot analyses of ZEB1 and USP1 in the skin of WT mice with Zer or NE-Zer treatment before UV irradiation.

Supplementary Material 2

## Data Availability

The datasets used and/or analyzed in the current study are available from the corresponding author upon reasonable request.
