# Supplementary material for: Interaction of TAGLN and USP1 promotes ZEB1 ubiquitination degradation in UV-induced skin photoaging
Source: Cell Biosci. 2023 May 6;13:80. doi: 10.1186/s13578-023-01029-z (PMC10163745; doi:10.1186/s13578-023-01029-z)
Supplement: Supplementary file 1 — Additional file 1: Fig. S1 a) Western blot analysis of USP1 expression after TAGLN overexpression. b) Duolink PLA demonstrates that USP1 interacts with TAGLN or ZEB1. c) Statistical analysis of Western blot results. d) Western blot analysis of ZEB1 protein expression after the co-knockdown of TAGLN and USP1. Fig. S2 SJB3-019A inhibited the deubiquitination of ZEB1 by USP1. Fig. S3 a) TIMP2 and NFKBIA mRNA levels quantified by q-PCR. b) Purified GST-tagged TAGLN was incubated with His-tagged USP1 in the presence or absence of the indicated amounts of Zerumbone. The interaction between TAGLN and USP1 was visualized using immunoblots. Fig. S4 Western blot analyses of ZEB1 and USP1 in the skin of WT mice with Zer or NE-Zer treatment before UV irradiation. [file 13578_2023_1029_MOESM1_ESM.docx]

Supporting Information for

## **Interaction of TAGLN and USP1 promotes ZEB1 ubiquitination degradation in UV-induced skin photoaging**

Yinan Li^1,2,*^, Xiu Huang^1,2,*^, Jing Jin^1,*^, Haohao Zhang^1,2,*^, Kai Yang^1^, Jingxia Han^1,2^, Ying Lv^2^, Yu Sun^2^, Cheng Yao^1,#^, Tingting Lin^3,#^, Caibin Zhu^1,#^, and Huijuan Liu^1,2,#^

^1^ Cheermore Cosmetic Dermatology Laboratory, Shanghai, China.

^2^ State Key Laboratory of Medicinal Chemical Biology and College of Pharmacy, Nankai University, Tianjin, China.

^3^ Medical plastic and cosmetic center, Tianjin Branch of National Clinical Research Center for Ocular Disease, Tianjin Medical University Eye Hospital, Tianjin, China.

^*^These authors have contributed equally to this work.

^#^Corresponding authors: correspondence and requests for materials should be addressed to Huijuan Liu (E-mail: liuhuijuanxyz@163.com), Tingting Lin (ltt6123@126.com), or Caibin Zhu (alex@cheermorecos.com).

**
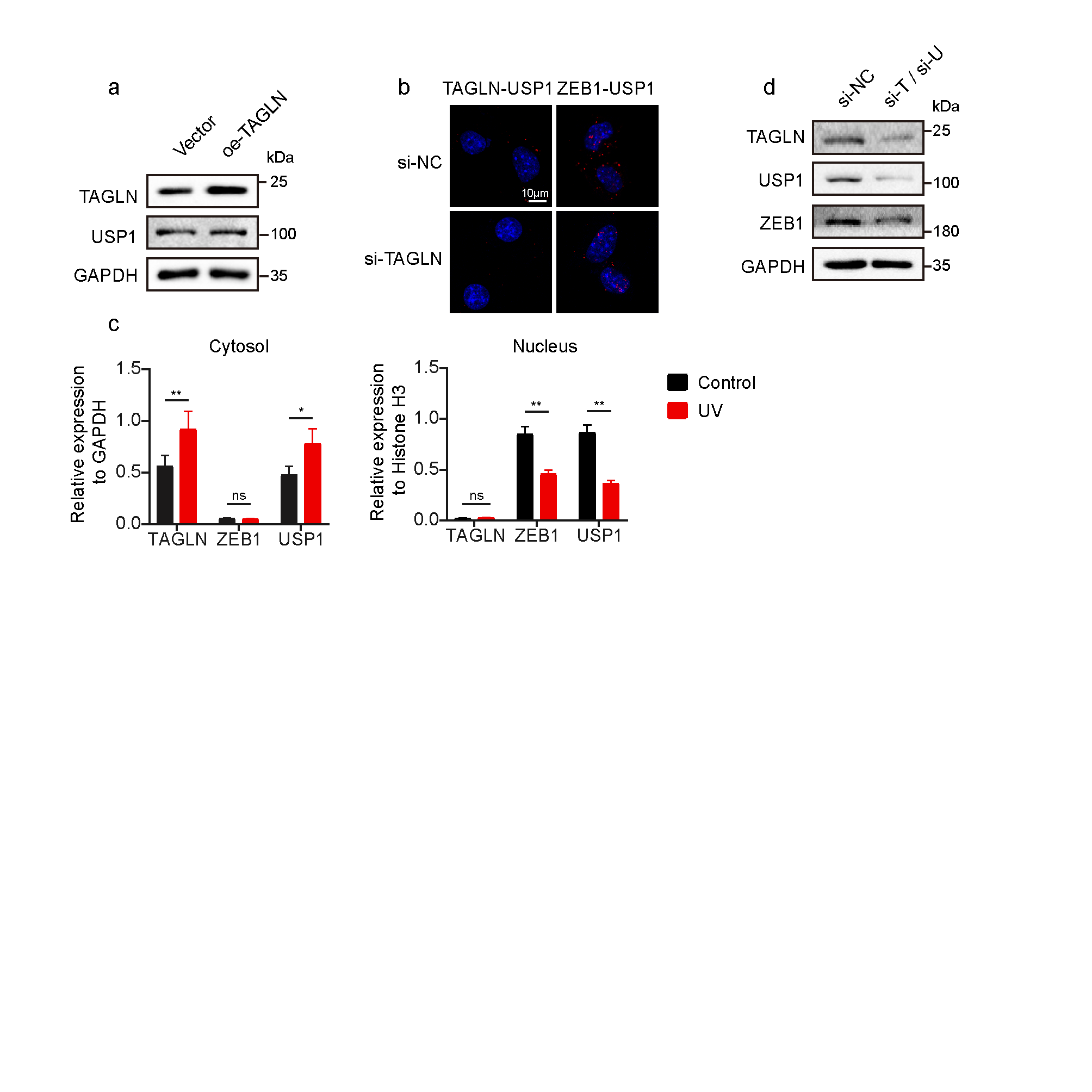
**

**Fig. S1** a) Western blot analysis of USP1 expression after TAGLN overexpression. b) Duolink PLA demonstrates that USP1 interacts with TAGLN or ZEB1. c) Statistical analysis of Western blot results. d) Western blot analysis of ZEB1 protein expression after the co-knockdown of TAGLN and USP1.

**
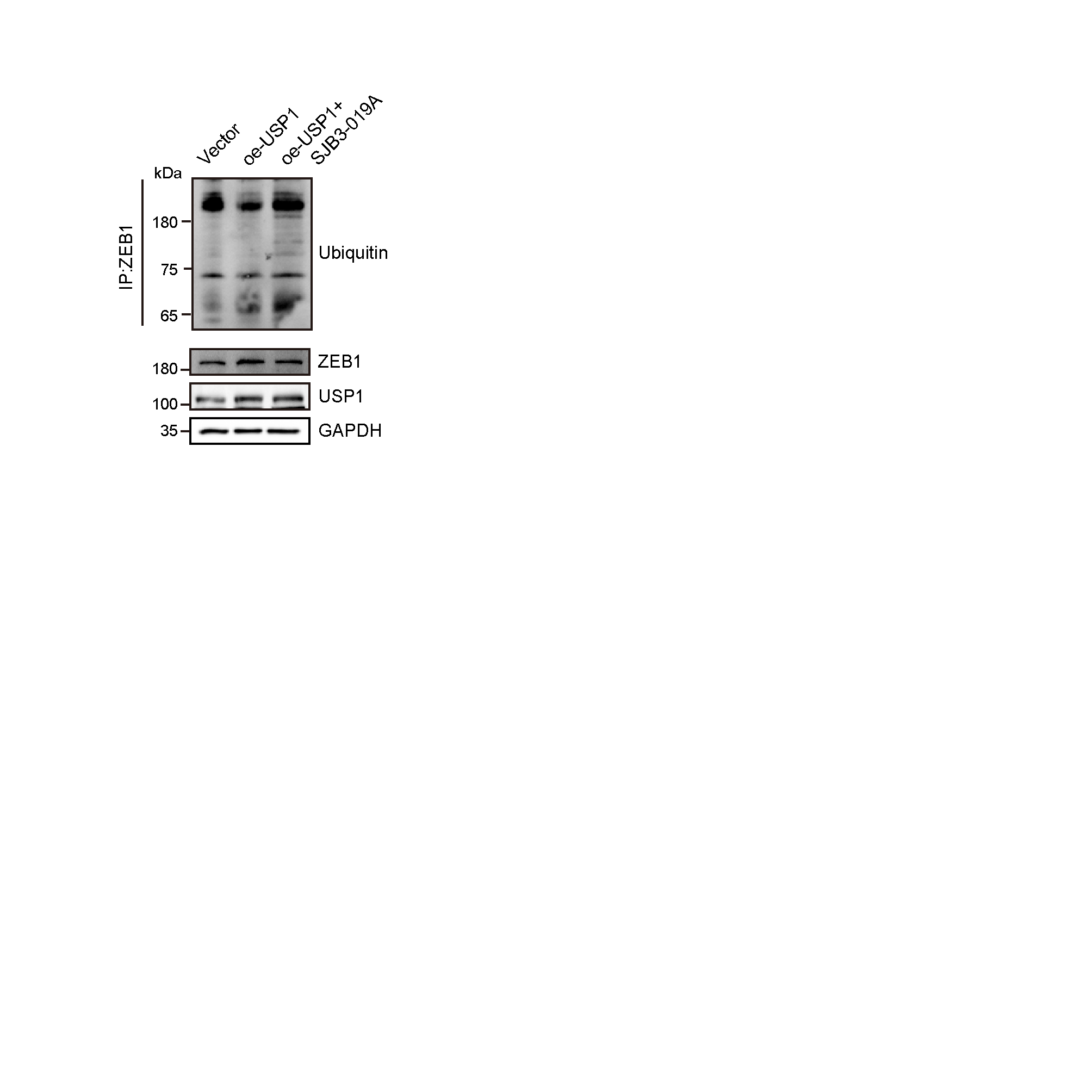
**

**Fig. S2** SJB3-019A inhibited the deubiquitination of ZEB1 by USP1.

**
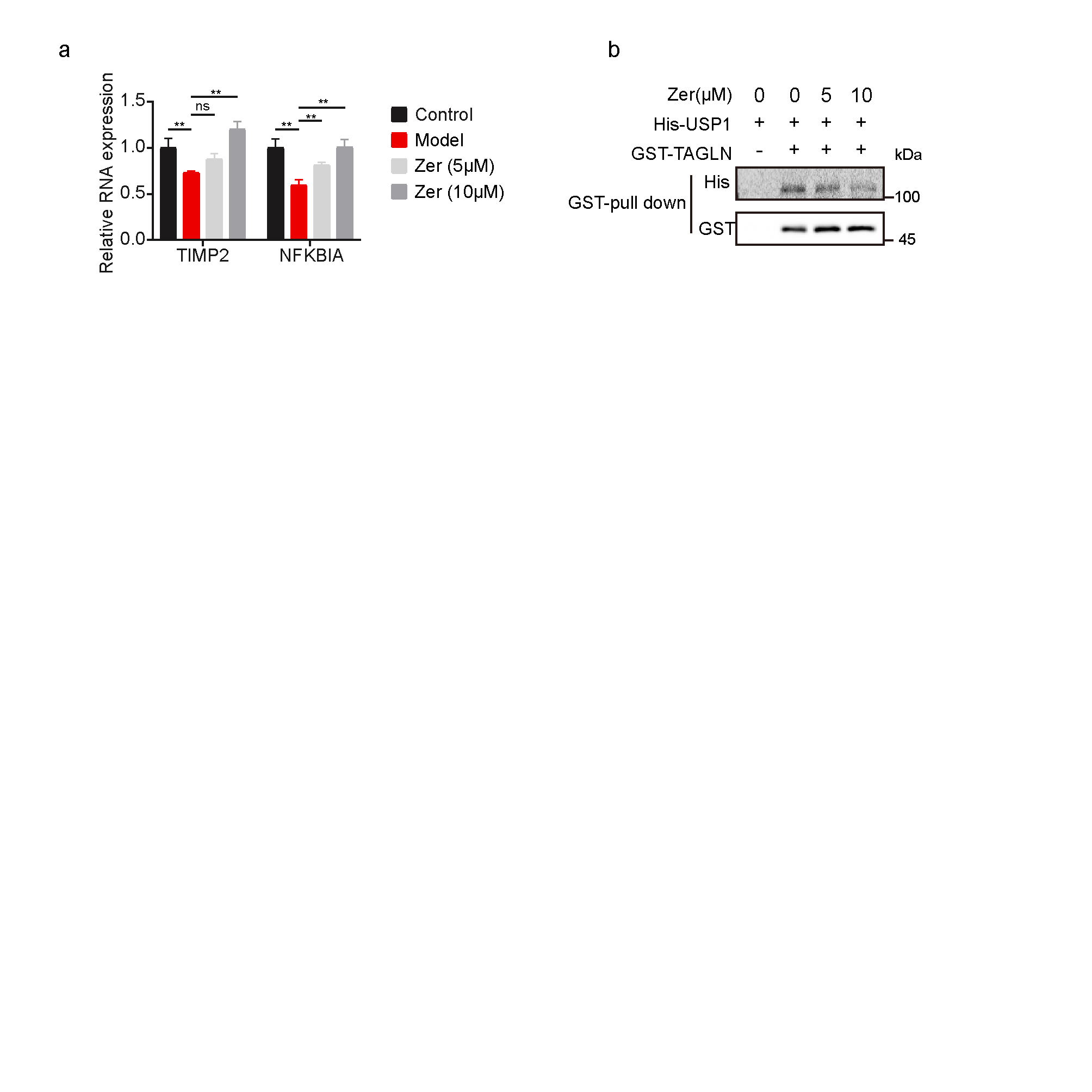
**

**Fig. S3** a) TIMP2 and NFKBIA mRNA levels quantified by q-PCR. b) Purified GST-tagged TAGLN was incubated with His-tagged USP1 in the presence or absence of the indicated amounts of Zerumbone. The interaction between TAGLN and USP1 was visualized using immunoblots.


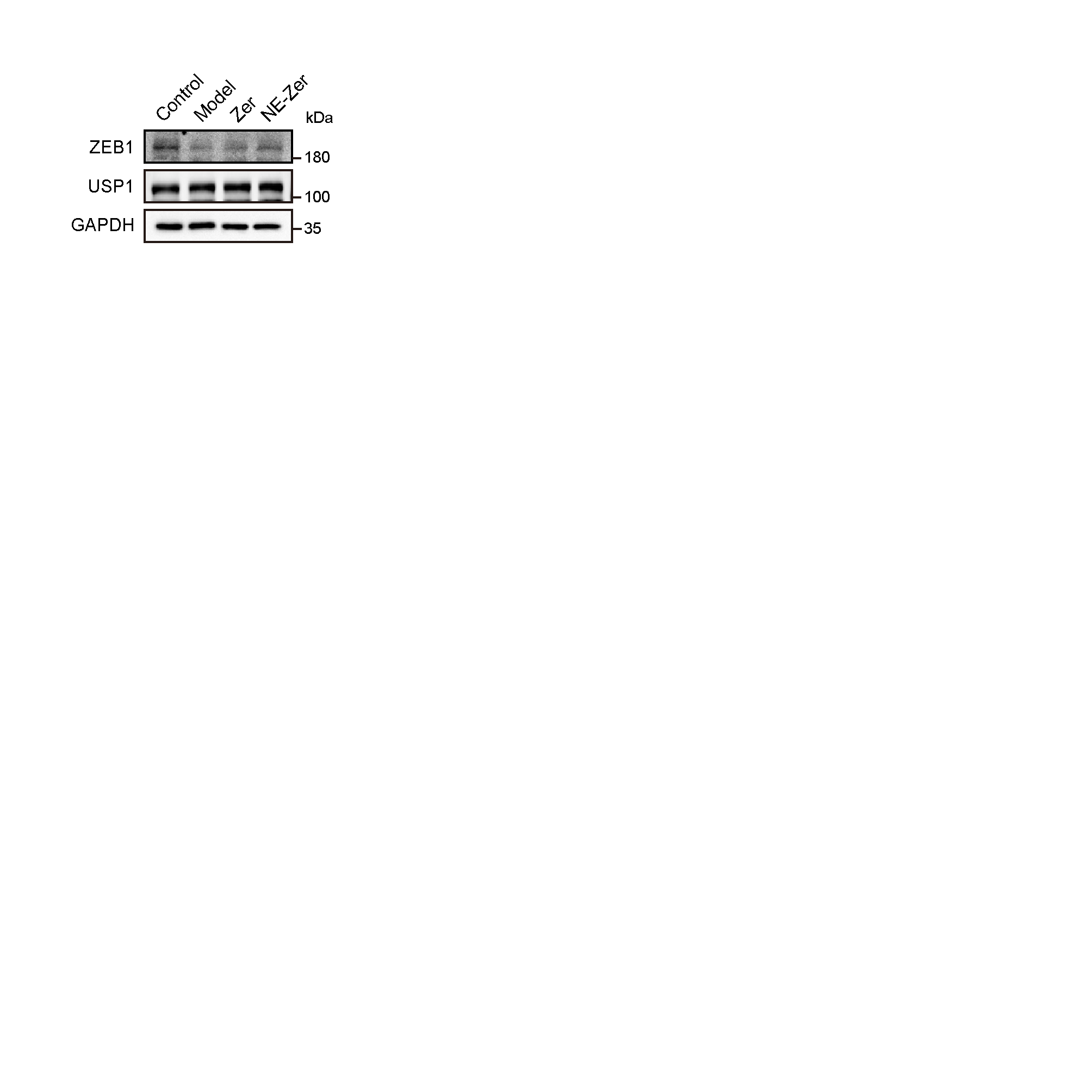


**Fig. S4** Western blot analyses of ZEB1 and USP1 in the skin of WT mice with Zer or NE-Zer treatment before UV irradiation.
