# Supplementary material for: Interaction of TAGLN and USP1 promotes ZEB1 ubiquitination degradation in UV-induced skin photoaging
Source: Cell Biosci. 2023 May 6;13:80. doi: 10.1186/s13578-023-01029-z (PMC10163745; doi:10.1186/s13578-023-01029-z)

Fig2b

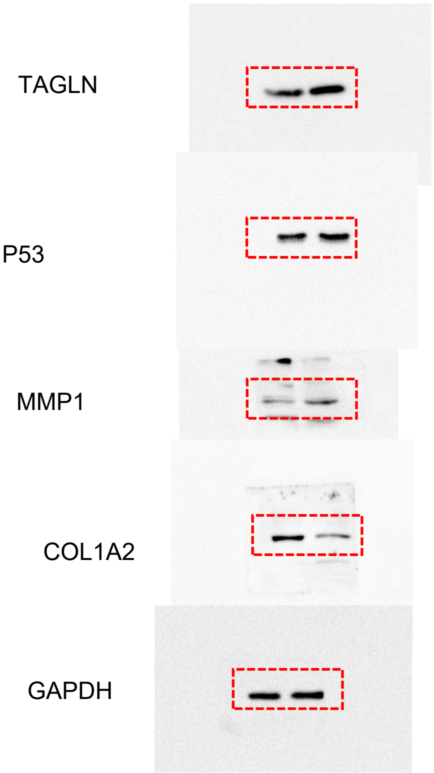

Fig2c

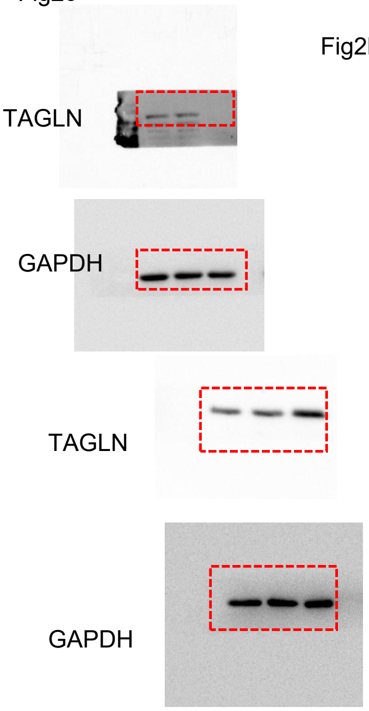

Fig2h

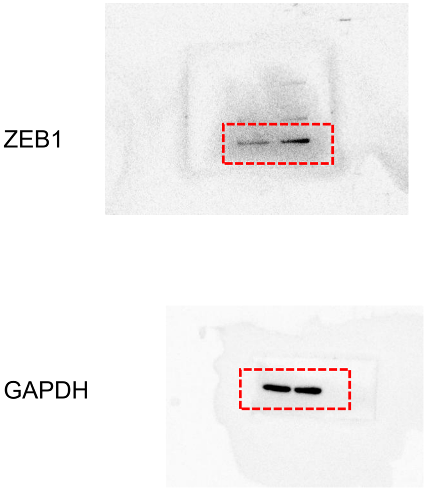

Fig2i

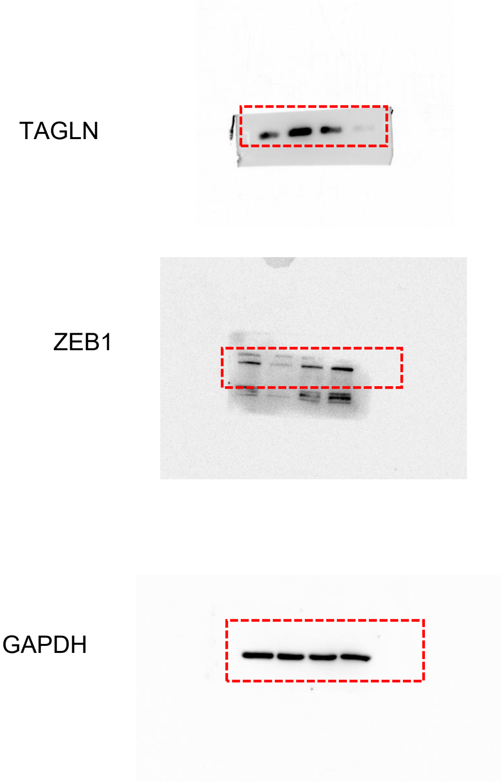

Fig2d

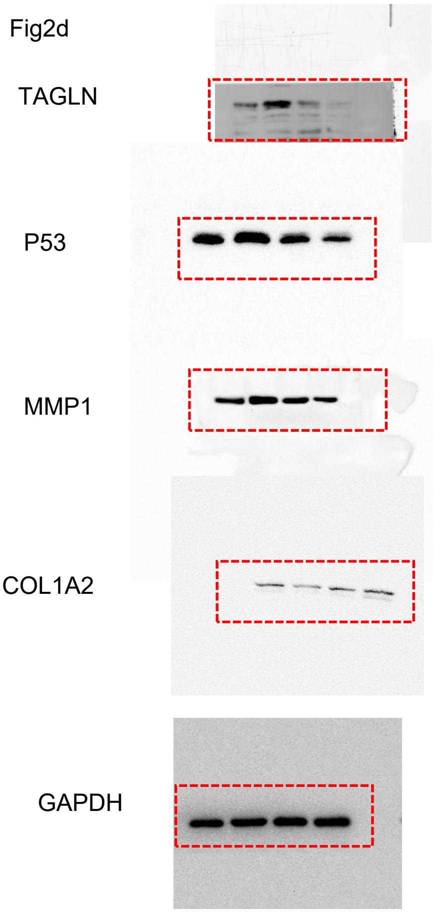

Fig3d

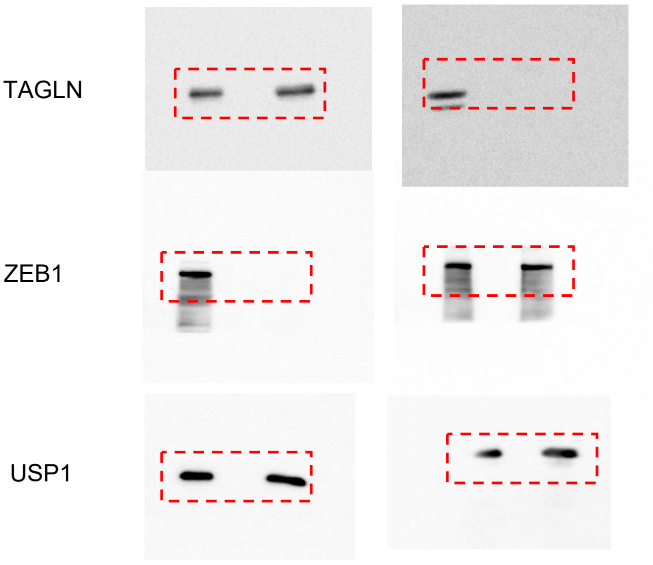

Fig3e

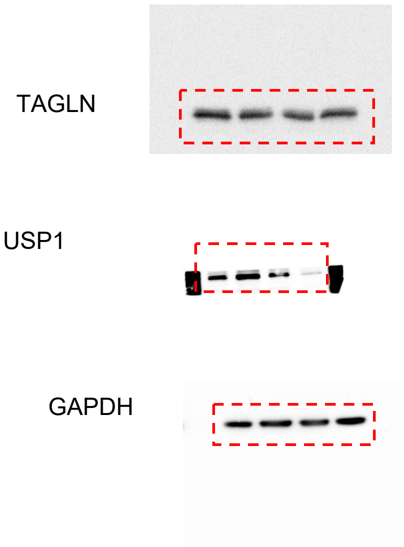

Fig3i

Fig3g

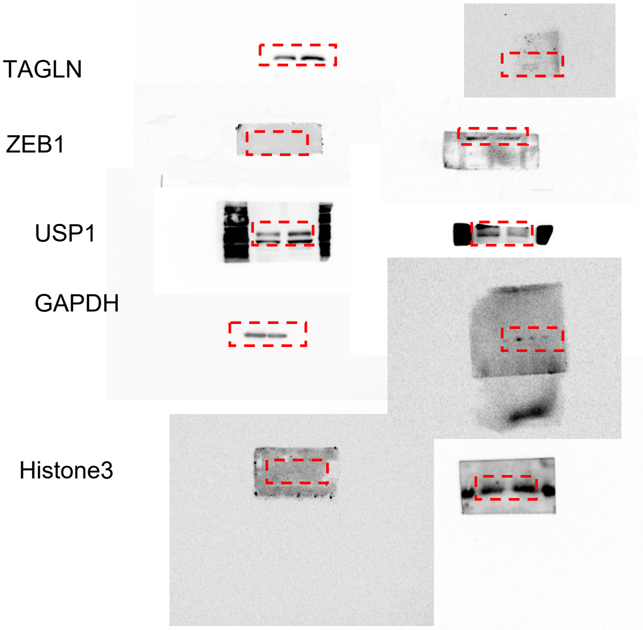

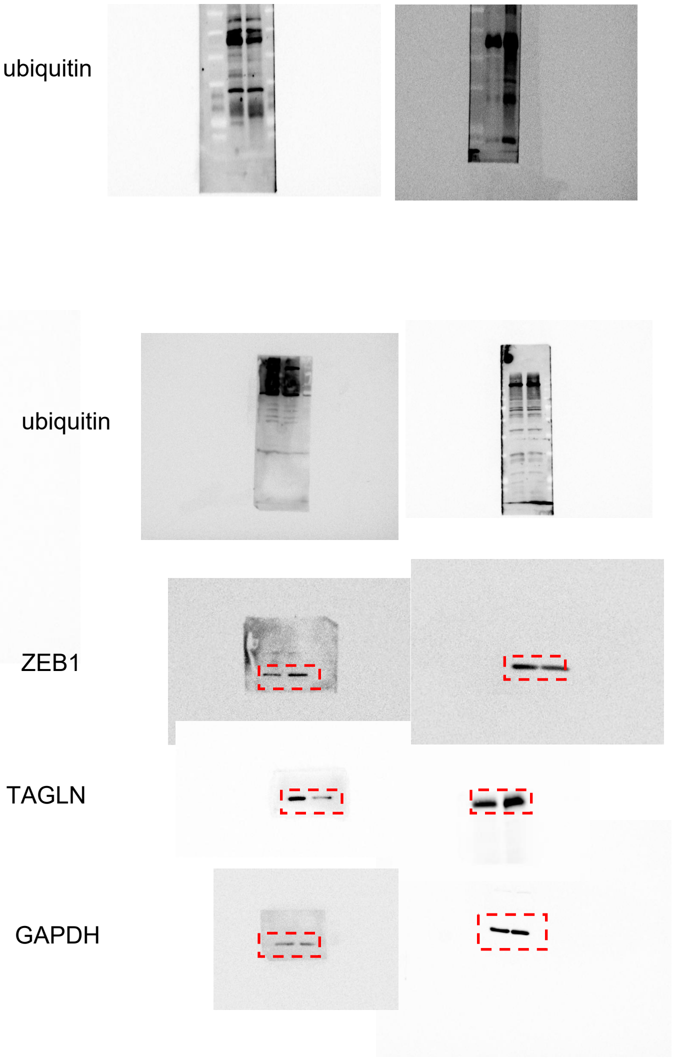

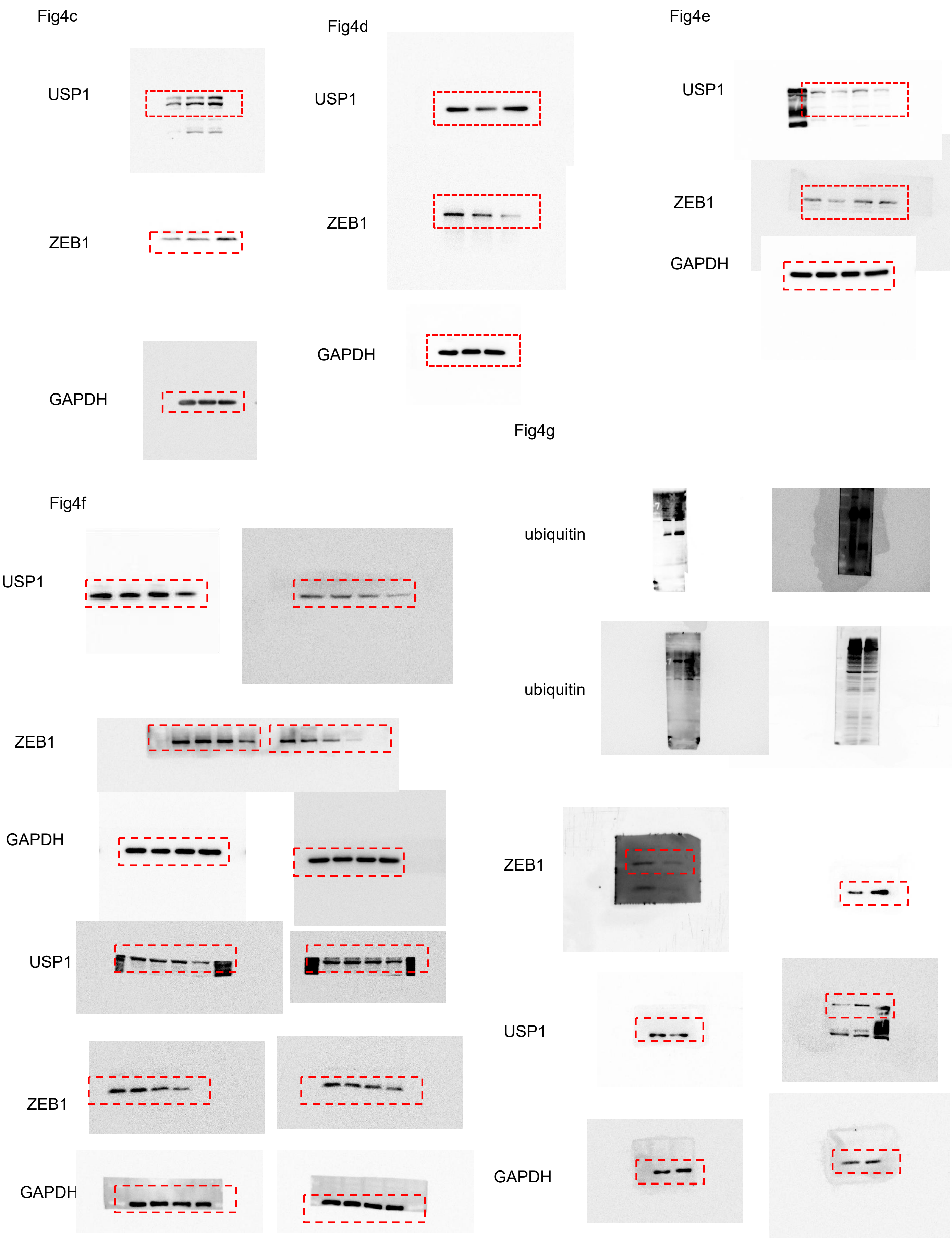
Fig4c
USP1
ZEB1
GAPDH
Fig4d
USP1
ZEB1
GAPDH
Fig4e
USP1
ZEB1
GAPDH
Fig4f
USP1
ZEB1
GAPDH
USP1
ZEB1
GAPDH
Fig4g
ubiquitin
ubiquitin
ZEB1
USP1
GAPDH

Fig5i

ZEB1

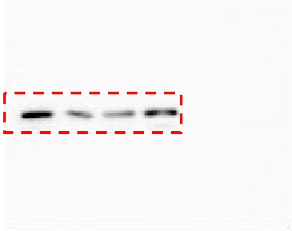

P53

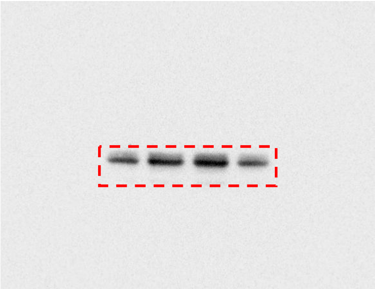

MMP1

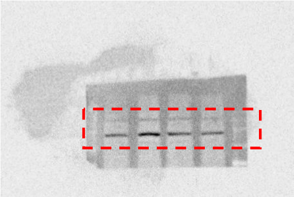

COL1A2

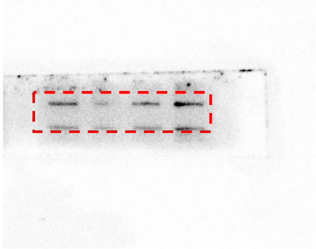

GAPDH

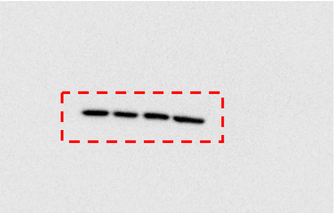

Fig5h

ubiquitin

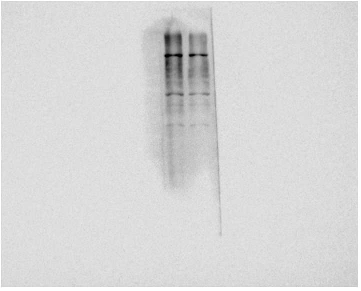

ubiquitin

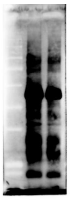

USP1

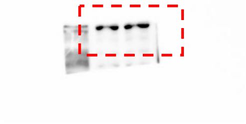

ZEB1

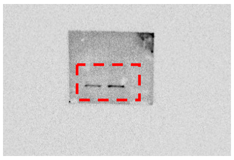

GAPDH

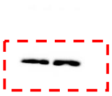

Supplement: Supplementary file 2 — Supplementary Material 2 [file 13578_2023_1029_MOESM2_ESM.pdf]
